# Quantitation of *Lupinus* spp. Quinolizidine Alkaloids by qNMR and Accelerated Debittering with a Resin-Based Protocol

**DOI:** 10.3390/molecules29030582

**Published:** 2024-01-24

**Authors:** Nikoleta Anna Madelou, Eleni Melliou, Prokopios Magiatis

**Affiliations:** Laboratory of Pharmacognosy and Natural Products Chemistry, Department of Pharmacy, National and Kapodistrian University of Athens, 15771 Athens, Greece; nikoletamadelou@gmail.com (N.A.M.); emelliou@pharm.uoa.gr (E.M.)

**Keywords:** *Lupinus* species, quinolizidine alkaloids, quantitation, qNMR, screening, lupin beans, resins

## Abstract

Quinolizidine alkaloids (QAs) are toxic secondary metabolites of the *Lupinus* species, the presence of which limits the expansion of lupin beans consumption, despite their high protein content. Evaluation of the level of alkaloids in edible *Lupinus* species is crucial from a food safety point of view. However, quantitation of QAs is complicated by the fact that not all important alkaloids used for quantitation are commercially available. In this context, we developed a method for the simultaneous quantitation of eight major lupin alkaloids using quantitative NMR spectroscopy (qNMR). Quantitation and analysis were performed in 15 different seed extracts of 11 *Lupinus* spp. some of which belonged to the same species, with different geographical origins and time of harvest, as well as in all aerial parts of *L. pilosus*. The mature seeds of *L. pilosus* were found to be a uniquely rich source of multiflorine. Additionally, we developed a protocol using adsorption or ionic resins for easy, fast, and efficient debittering of the lupine seeds. The protocol was applied to *L*. *albus*, leading to a decrease of the time required for alkaloids removal as well as water consumption and to a method for QA isolation from the debittering wastewater.

## 1. Introduction

The *Lupinus* genus is one of the most diverse and widespread taxonomic groups of flowering plants, including both annual and perennial species [1,2]. It belongs to the Fabaceae family and was introduced by Linnaeus in his work “Species Plantarum”. Most species occur in North America, with two main centers of species diversity present in western North America (c. 100 species) and the Andes (c. 85 species) [3,4]. In contrast, only 13 species are found in the Old World, predominantly around the Mediterranean. There is, however, a significant discrepancy in the literature regarding the number of representatives of the genus. According to C. P. Smith’s book, at least 500 species *Lupinorum*, as he called them, had been described up to that time. The same number is also referred to by Wink et al. [5], while Gresta et al. [6] listed only 170 species and Reinhard et al. [7] over 400. Similar differences are found in many more studies.

Lupin species are divided into Old (OWL: Old World Lupins) and New World (NWL: New World Lupins). OWLs are represented by at least 12 species (13 according to some studies) while NWLs include several hundred species, annual or perennial with compound or simple leaves. In contrast, only annual plants with compound leaves appear in the OWL [2,6]. The latter have been divided, depending on the texture of the sperm, into two groups by Plitman and Heyn [8], into Malacospermae and Scabrispermae.

Wild representatives of the genus occur in North and South America, the Mediterranean region, and North Africa. During the early period of colonization, many of them were introduced to southern Africa and Australia. Today, they are better known as ornamental plants and have been studied for their agronomic characteristics and nutritional value as they contain 40% of protein. In most parts of the world, lupins have been grown for animal feed, as a green manure and less for human consumption due to their bitter alkaloids that need to be removed [2,9,10]. There are four cultivated species worldwide, *L. albus*, *L. angustifolius*, *L. mutabilis, and L. luteus* [9]. Despite the measurable progress in domestication and the provision of lupins as a source of high-protein feed and food product, they are still considered as a neglected crop on the worldwide scale.

**Figure 1:**
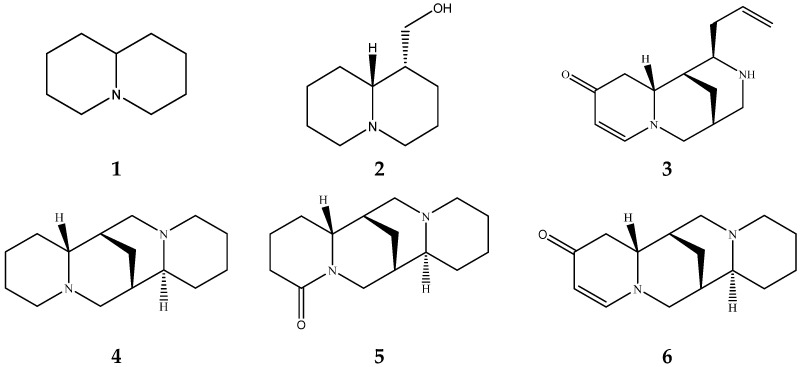

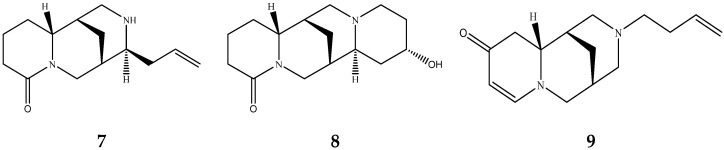
Quinolizidine core structure and studied quinolizidine alkaloids found in *Lupinus* species (**1**) quinolizidine core, (**2**) lupinine, (**3**) (−)−albine, (**4**) sparteine, (**5**) lupanine, (**6**) multiflorine, (**7**) angustifoline, (**8**) 13-OH-lupanine, (**9**) 11,12-seco-12,13-didehydromultiflorine.

Lupin has been recognized as a highly nutritious grain, providing a high quantity of proteins compared to traditional legumes, as well as high content of essential fatty acids and dietary fiber. The seeds are often consumed after cooking as whole seeds, but they can also be used as food ingredients (flour) in the production of bread, gluten-free cakes, or dairy products. In addition to nutrients, lupin seeds contain secondary metabolites such as polyphenols, carotenoids, phytosterols and, most interestingly, bitter quinolizidine alkaloids (QAs) (Figure 1). QAs have been reported to possess various pharmacological effects such as anticonvulsant, anti-inflammatory, antiviral, antitumor, antipyretic, antihepatitic B, antifibrotic, antiallergic, antidiarrheal, analgesic, and antimicrobial [11,12,13]. Several in vivo studies have been conducted to elucidate antihyperglycemic properties of QAs. Kubo et al. focused on one among the pharmacologically active lupin alkaloids, multiflorine, and demonstrated that a single dose acutely improves glucose tolerance in mice. Based on this finding, they successfully designed several derivatives of multiflorine with robust glucose lowering activities [14,15,16,17]. In the pancreas, sparteine induces insulin and glucagon secretion and exhibits a hypoglycemic effect. Sparteine has also demonstrated protective activity against diabetes-associated DNA damage [18]. Lupanine potentiates glucose-stimulated insulin release by directly affecting K_ATP_ channels [19,20]. Overall, sparteine and lupanine are the most studied QAs, while cytisine is well established in Eastern Europe and Canada for smoking cessation [21]. Moreover, all alkaloids occurring in *Lupinus* have both biological and ecological significance and also considered to be taxonomical markers [22]. Another relevant function of QAs is their use as fertilizers for some crops. Also, pure QAs or mixtures of plant extract can be used to protect plants against noxious insects [23]. It is crucial to note that comprehending the conformational structure of molecules is essential for gaining insights into various biological processes, such as pharmacological activity and taste perception. Recent research on the hypoglycemic impact of certain quinolizidine alkaloids indicates that compounds with more labile structures may exhibit higher effectiveness. Additionally, the taste intensity of bis-quinolizidine compounds appears to be related to their conformational equilibrium state [24].

Most published debittering processes include a soaking stage of the seed [12,25]. A soaking stage is important because it increases the water content of the seed and facilitates the extraction of alkaloids in subsequent stages. The cooking stage is essential to inactivate the germination capacity of the seeds, their enzymes (lipase, lipoxygenase), to eliminate occurring microorganisms for food safety, to reduce the loss of proteins through their coagulation, and to facilitate the leaching of the alkaloids by increasing the cell wall permeability. One alternative way for reducing the concentration of alkaloids is microbial debittering. Debittering of lupin seeds using water has been used since pre-Inca times in the Andean Region. The residual levels of alkaloids have traditionally been verified based on presence or absence of bitter taste only [26]. A recently published article, aiming to develop a novel lupin debittering method used ultrasound in different temperatures, as an alternative to the traditional method (including a boiling stage in water for 75 min followed by soaking for 144 h) [27].

Most articles about the analysis and quantitation of QAs use gas chromatography equipped with a flame ionization detection (GC-FID) [28,29] or mass spectrometry (GC-MS) [7,30,31,32,33,34], while HPLC application for that purpose is extremely limited [35,36,37]. For instance, a GC-MS based method was developed from Resta et al. using lupanine as external standard in samples of lupin-based products [31] and from Romeo et al. using sparteine [33]. The quantitation of *Lupinus* alkaloids is complicated by the fact that commercial standards are not easily available. Lupanine, lupinine, and sparteine are available from a few different suppliers, but the other QAs are either available from a single source only or not available at all. Isotopically labelled analogues are also not available. Due to the lack of available reference standards, quantitation is often performed by comparison of relative peak areas to that of an available standard, e.g., sparteine or lupanine [12,31,33]. This may introduce considerable uncertainty in the reported concentrations [31]. A recent article reported a HPLC-MS/MS method development, applied to four different batches of raw seeds of *L. albus* for the quantitation of thirteen alkaloids. From all thirteen QAs standards studied, only six were identified and quantified [35].

The main objective of the current work was to develop an easy, fast, and efficient method for quinolizidine alkaloids quantitation based on qNMR spectroscopy that could be used to monitor the levels of the QAs during a new debittering process involving adsorption and ionic resins. In addition, we aimed to identify species that could be rich sources of specific alkaloids and in parallel we investigated the possible differences between various cultivated and wild *Lupinus* spp., and variations among different parts of the plants.

## 2. Results

### 2.1. ^1^H-NMR Peak Assignment

**Figure 2:**
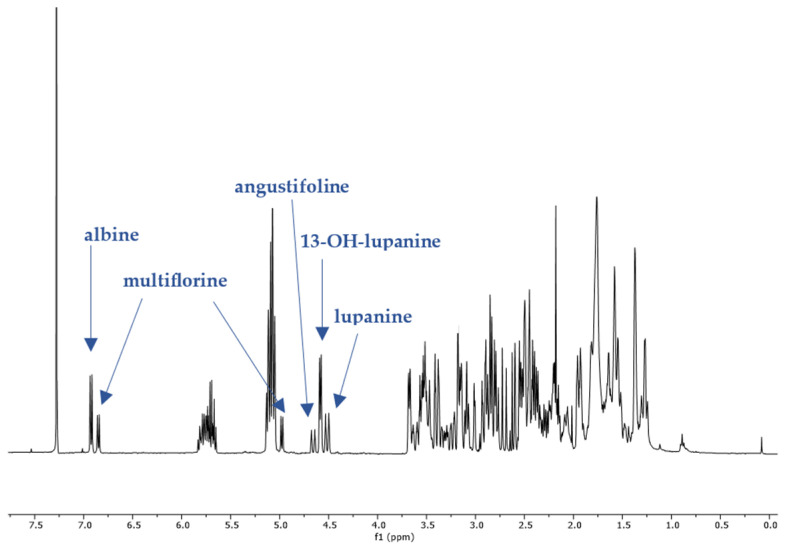
^1^H-NMR spectrum (400 MHz, CDCl_3_, δ-values in ppm) from a fraction of an extract of *L. albus* obtained during column chromatography, showing the characteristic peaks of five studied QAs. Line arrows indicate the corresponding peaks used for quantitation of albine, multiflorine, angustifoline, 13-OH-lupanine and lupanine (peak heights are not representative of the concentrations in the original plant extract).

**Figure 3:**
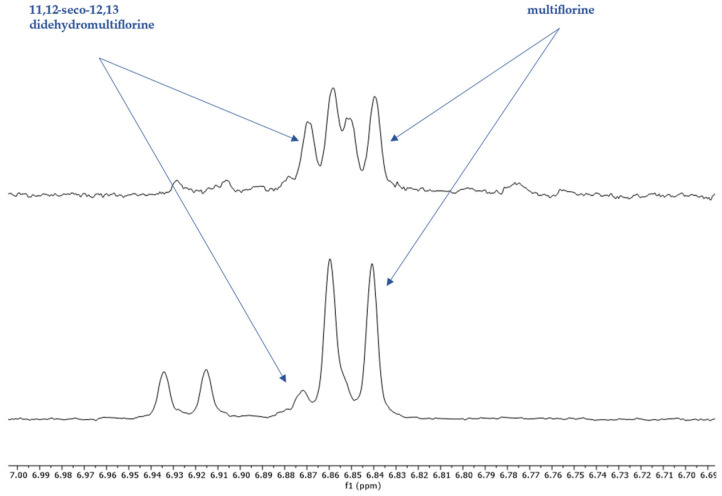
^1^H-NMR spectrum (400 MHz, CDCl_3_, δ-values in ppm) from *L. albus* extracts, showing overlapping signals of 11,12-seco-12,13 didehydromultiflorine with multiflorine.

The non-overlapping peaks that could be used for quantitation of each QAs are presented in Table 1 and Table S1 and a characteristic spectrum of their mixture is presented in Figure 2. More specifically, observation of the ^1^H-NMR spectrum of lupanine revealed that α-diastereotopic proton H10 was the most de-shielded proton of the molecule, due to the negative inductive effect of nitrogen as well as an additional de-shielding by the carbonyl group. In particular, H10eq proton appeared at chemical shift δ 4.49 as a doublet of triplet peak, with a coupling constant of 13.29 and 2.3 Hz. A doublet peak of 13-OH-lupanine was observed at 4.58 ppm, a doublet for multiflorine at 6.84 ppm with a coupling constant of 7.7 Hz, a doublet for 11,12-seco-12,13-didehydromultiflorine at 6.87 ppm with a coupling constant of 7.36 Hz, a doublet of triplet for angustifoline at 4.66 ppm with a coupling constant of 13.6 and 2.1 Hz, a doublet for albine at 6.92 ppm with a coupling constant of 7.53 Hz and a doublet of doublet for lupinine with a coupling constant of 10.45 and 4.12 Hz. All above-mentioned peaks integrate for one proton. A more detailed discrimination between the quantitation peaks of multiflorine and 11,12-seco-12,13-didehydromultiflorine are shown in Figure 3. In order to quantitate those two alkaloids in plant extracts, only one scale of each double peak was used. In a few samples, we were also able to observe a doublet of pentaplets for sparteine at 2.80 ppm with a coupling constant of 9.8 Hz, However, in most cases this peak was overlapping and was not useful for quantitation.

### 2.2. Differences among Species and Origins

The concentrations of the eight quantitated quinolizidine alkaloids lupanine,13-OH-lupanine, multiflorine, lupinine, 11,12-seco-12,13 didehydromultiflorine, albine, angustifoline and sparteine in the extracts of the seeds of each of the studied species are presented in Table 2 and Figure S1. The corresponding NMR spectra of all the samples are presented in Figure S2.

**Figure 4:**
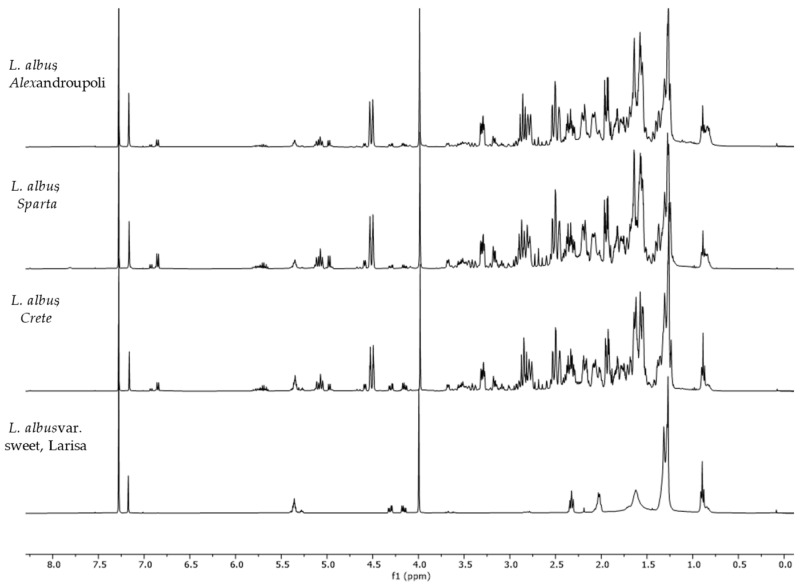
^1^H-NMR spectra (400 MHz, CDCl_3_, δ-values in ppm) from *L. albus* spp. originating from Crete, Alexandroupoli, Sparta and the sweet variety from Larisa.

Among the studied species, *L. albus* from Sparta showed the highest content of QAs. In contrast, the sweet variety of *L. albus* showed an almost complete lack of alkaloids apart from traces of multiflorine. The ^1^H-NMR spectra of *L. albus* (originating from three distinct regions of Greece: Crete, Alexandroupoli, Sparta) and the sweet variety originating from Larisa are presented in Figure 4, providing an example of the differentiation among sweet and bitter varieties of the same species. The results obtained for each of *L. albus* crops showed variation in alkaloids concentration among the different geographical origins. Lupanine was the most abundant alkaloid in all *L. albus* (Sparta, Alexandroupoli, Crete), but the plants collected in Sparta showed the highest concentration both in QAs and lupanine. *L. luteus* was the sample with the lowest alkaloids concentration, as no evidence of alkaloids were detected with ^1^H-NMR.

**Figure 5:**
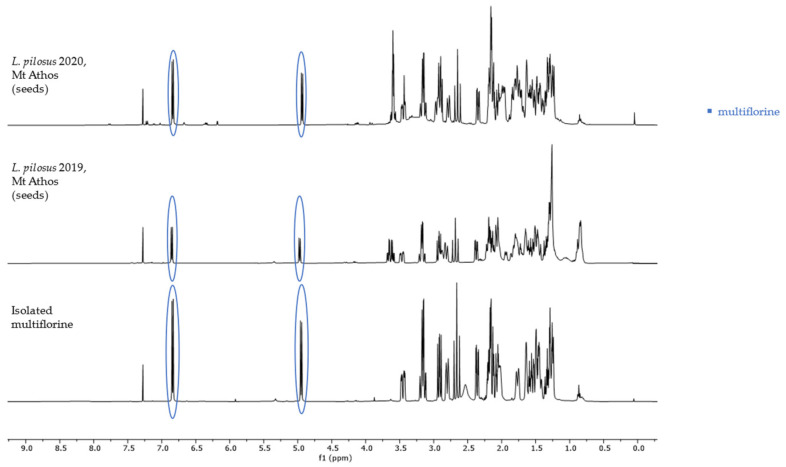
^1^H-NMR spectra (400 MHz, CDCl_3_, δ-values in ppm) from isolated multiflorine and *L. pilosus* mature seed extracts from Mt Athos in two consecutive years.

Interestingly, the mature seeds of *L. pilosus* were found to be a uniquely rich source of almost pure multiflorine accounting for 97% of total alkaloids (6.83 mg/g) and only a small percentage of lupinine 3% (0.23 mg/g) (Figure 5). Multiflorine was detected in only two species: *L. albus* and *L. pilosus* in concentrations 0.16–2.49 mg/g and 6.83 mg/g, respectively.

Albine and 11,12-seco-12,13 didehydromultiflorine were only detected in *L. albus* among the studied species, while lupinine was only detected in *L. pilosus*. Lupanine was the predominant alkaloid, with the highest concentration (22.55 mg/g) in *L. albus* from Sparta, while angustifoline was the major alkaloid (1.00 mg/g and 2.22 mg/g) in *L. angustifolius* and *L. perennis*, respectively.

### 2.3. Differences among Plant Parts

This is the first comparative analysis for all aerial parts of *L. pilosus*, since only leaves and seeds have been studied previously [5,38]. In this study, aerial parts of *L. pilosus* from Crete were collected in an early stage of development (20 days after flowering), separated into flowers, leaves, pods, fruit beans and stems and subjected to chemical analysis. Results are presented on Table 3 and the corresponding NMR spectra in Figure S3.

In stems, lupanine (dt at 4.49 ppm) was the only alkaloid present, while in flowers the only one was multiflorine (d at 6.84 ppm and 4.95 ppm). Leaves had multiflorine as the predominant alkaloid, with traces of lupanine. In pods, multiflorine was the predominant alkaloid with second lupanine and significant amounts of albine, angustifoline, 11,12-seco-12,13 didehydromultiflorine and 13-OH-lupanine. In the immature seeds the main alkaloid was lupanine, with significant amounts of the other four QAs. In contrast, with the mature seeds reported in 2.2 only multiflorine was detected. Lupinine was completely absent from all plant parts. Results were also confirmed with GC-MS analysis.

### 2.4. Resin Debittering Process

**Figure 6:**
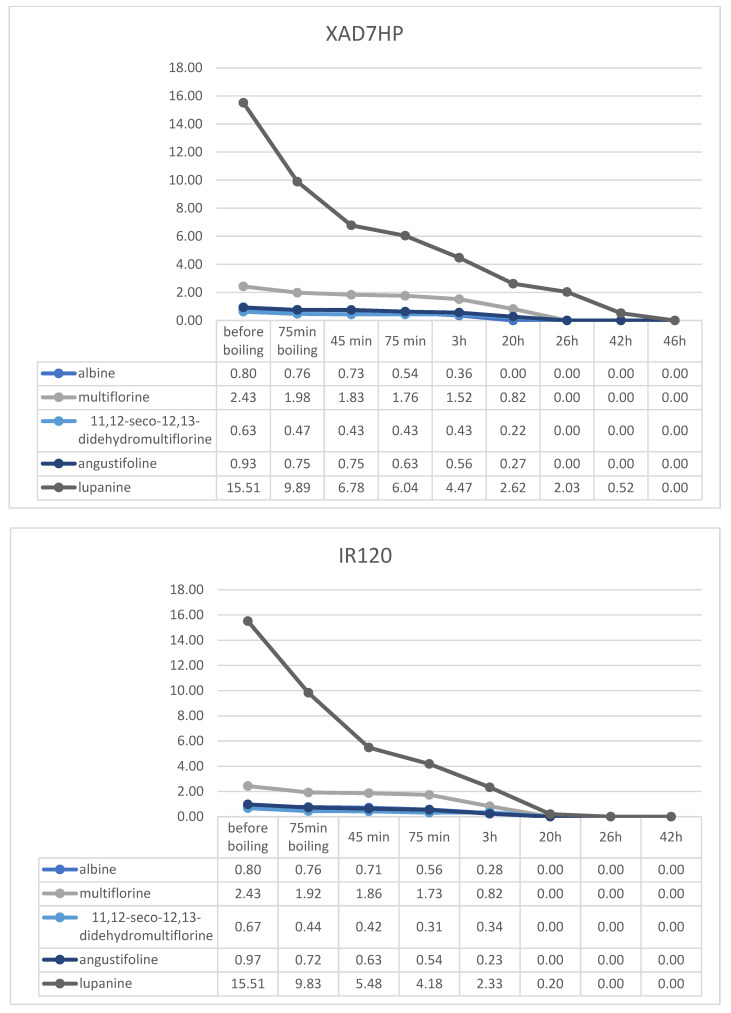

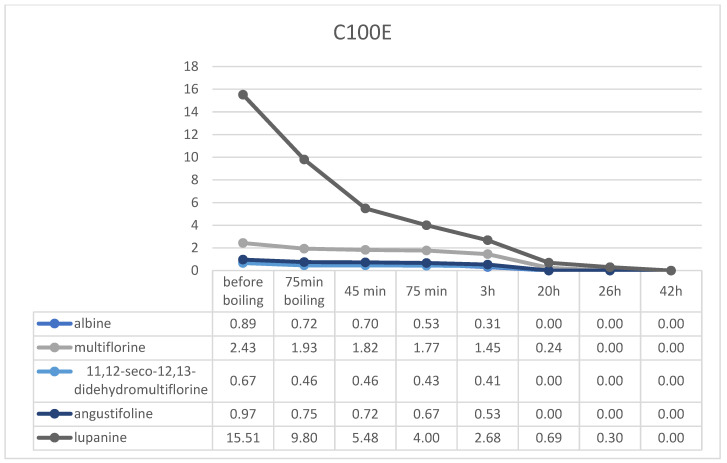
Concentrations of QAs in *L. albus* seeds during the application of the debittering protocol with each of the studied resins, expressed in mg/g DW.

^1^H-NMR quantitation of alkaloids in lupine seeds during the resin debittering process was performed following the same quantitation protocol that was developed for the unprocessed seeds. Samples of lupins were quantified before the beginning of the debittering process, after boiling, after 45, 75 min, 3, 20, 26, 42 and 46 h of debittering. Diagrams showing the removal rate of alkaloids in each resin as well as the concentration of them are presented in Figure 6. Results are presented in mg/g DM.

The resin which showed the highest retainment of alkaloids was the ion exchange resin IR120, since almost all alkaloids were eliminated after 20 h, except for 0.2 mg/g lupanine. Also, ion exchange C100E, which is suitable for food processing, presented strong retention, with alkaloids elimination being achieved in 26 h. Finally, in the case of the adsorptive non-ionic XAD7HP, after treatment for 42 h, only 0.5 mg/g lupanine remained in the seeds.

### 2.5. QAs Recovery from Resins

**Figure 7:**
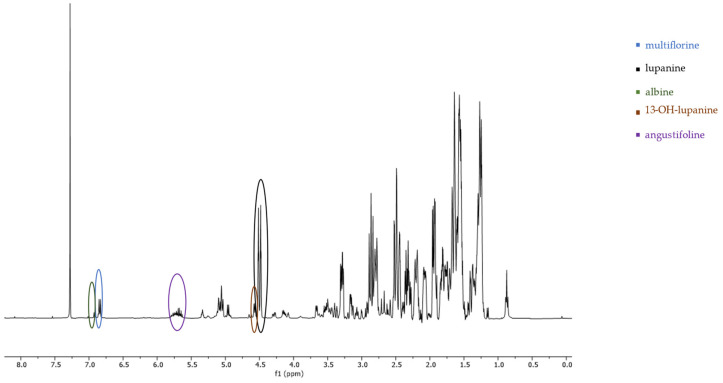
^1^H-NMR spectra (400 MHz, CDCl_3_, δ-values in ppm) of QAs recovered from Purolite C100E debittering water.

The recovery of QAs from the anionic resins was performed with three different washing solutions: NaOH 10% (*w*/*w*) in water, NaCl 10% (*w*/*w*) in water, and water. The recovery from the adsorption resin was performed using EtOH. The treatment of the acidic resins with NaOH in water was found to be the most efficient method. At the end, the NaOH solution was extracted with CH_2_Cl_2_ which, after evaporation, afforded the QAs in dry form (Figure 7).

## 3. Discussion

The main objective of this work was to develop an easy and efficient method for quinolizidine alkaloids quantitation that could be used to investigate the possible differences among various *Lupinus* spp. both cultivated and wild. Based on the previous successful use of ^1^H-NMR for the qualitative and quantitative characterization of bioactive ingredients in olive oil, wine, and beer, we applied this method for screening of alkaloids profile on selected *Lupinus* extracts. Although the target analytes (lupanine, multiflorine, albine, angustifoline, lupinine, 13-hydroxylupanine, sparteine, and 11,12-seco-12,13-didehydromultiflorine) have been previously analyzed mainly using gas chromatographic methods, NMR had been used only for qualitative purposes. Regarding the gas chromatography methods, the time for analysis of the extracts is significantly longer than the proposed method in the present study especially when a derivatization step is applied. Although the derivatization step is not always necessary for analysis of lupin seeds, it is sometimes used to obtain lower LODs, particularly in complex matrices, such as food. Moreover, all chromatographic methods need standards, construction of calibration curves and repetitive calibration leading to longer analysis time, especially when numerous samples need to be analyzed. Quantitation of QAs is also complicated by the fact that not all-important alkaloids used for quantitation are commercially available. So, we focused on the development of a method that does not need separation of the analytes before quantitation to avoid several time-consuming procedures.

The qNMR method presents distinct advantages over chromatographic approaches commonly applied for the analysis and quantitation of quinolizidine alkaloids in *Lupinus* spp. products or seeds. Historically, the quantitation of QAs has relied on chromatographic methods, which, although effective, entail prolonged analysis times and extensive extraction or pretreatment procedures [7,28,29,30,31,32,33,34,35,36,37,39]. Analytical methods based on capillary high resolution gas chromatography have been in widespread use since the early 1980s, with minimal evolution over time. Liquid chromatography-based methods are not often used for the determination of QAs. As previously indicated, both chromatographic techniques are complicated by the limited availability and high cost reference standards. This underscores the advantages presented by the qNMR method. Specifically, the developed method requires a total time of less than 4 min per sample, without long pretreatment procedures. In all existing chromatographic methods (GC-MS, GC-FID, UPLC-MS/MS, HPLC-MS/MS, or LC-MS/MS) the time of analysis ranges from 15 min to 40 min, without considering pretreatment process, analysis of standard solutions or/and analysis of standard spiked samples.

The obtained ^1^H-NMR spectra of all studied extracts yielded well-resolved peaks without overlapping, except sparteine which could be clearly observed only in a few samples. The lack of overlapping was confirmed by two-dimensional heteronuclear single-quantum correlation (2D HSQC) spectra. Thus, the methodology proved to be sensitive to detect differences in bioactive substances content among and within *Lupinus* species. The method was applied for screening the QAs in the seeds of 11 *Lupinus* species. The main component of all the studied *Lupinus* species appeared to be lupanine, except for the mature seeds of *L. pilosus*, in which the main component was multiflorine. This finding is of particular interest because *L. pilosus* seeds at the appropriate time of harvest could be useful a unique source of almost pure multiflorine.

Moreover, an array of analyses was conducted on samples originating from diverse geographical locations for the two principal representatives of the plant in Greece (*L*. *pilosus* and *L. albus*). Additionally, a comprehensive examination was undertaken on all aerial parts of *L. pilosus*, a so far unstudied species. Intriguingly, a comparative assessment of flowers, leaves, and stems derived from the same *L. pilosus* plant revealed both qualitative and quantitative distinctions in the corresponding herbal extracts. As anticipated, the stems exhibited the lowest concentration of QAs, whereas leaves, flowers, pods, and fruit beans exhibited progressively higher concentrations. Furthermore, flowers exclusively contained multiflorine, leaves contained both lupanine and multiflorane, and the rest of plant parts appeared a variation of four more QAs.

Alkaloids content in plants may increase with treatments high in nitrogen. There are, however, many exceptions to this. Overall, it does seem that alkaloids content is generally related to the nitrogen levels available to plants. Two basic factors seem to influence this relation: (1) the biosynthetic nature of alkaloids themselves and (2) the balance of nitrogen and other nutrients in the soil. High or low concentrations of nitrogen in soil seem to influence alkaloid content in the plant despite the biosynthetic nature of alkaloids. In poor in nitrogen environments, plants suffer from nutritional stress and the production of alkaloids seems to increase [22]. Early drought stress tends to increase the alkaloid content, whereas terminal stress has the opposite effect; these effects are mainly observed when stress was present during the vegetative growth stage [40]. This variability is related with the pedoclimatic conditions and the geographic origin of the lupin plants and is a potential hindrance to the wider use of lupin for human consumption and a toxicological topic for consumers safety. Furthermore, a new debittering technique for extracting and processing lupine alkaloids was developed. This process effectively removed QAs from the seeds, making them edible in only 26 h. In general, lupine debittering techniques can be thermal, aqueous, alkaline, and/or acidic. According to literature data, only aqueous processes are applied to lupins intended for human consumption, which takes 90–140 h until alkaloids removal. These techniques also require large amounts of water and are therefore not environmentally friendly. However, the developed debittering process decreased the time required for alkaloids removal about 4 times and at the same time decreased dramatically the water consumption and could be easily adopted by the industry to reduce time and costs of lupin debittering.

## 4. Materials and Methods

### 4.1. General

All solvents were of analytical grade (Merck). Syringaldehyde (98% purity, Sigma-Aldrich, Steinem, Germany) was used as internal standard (IS). IS solution was prepared in acetonitrile at a concentration of 0.5 mg/mL and kept at 4 °C. The IS solution was left to reach room temperature prior to use. The quantitative determination of quinolizidine alkaloids was performed using NMR spectroscopy with a Bruker Avance DRX 400 MHz (National and Kapodistrian University of Athens, Greece). The ^1^H-NMR spectra were processed using either the MNova 6.0.2 (Mestrelab Research, Santiago De Compostela, Spain) or the TOPSPIN 4.1.4. software (Bruker, Billerica, MA, USA).

### 4.2. Plant Material and Processed Products

A total of ten commercially available lupins, including wild *L. perennis*, were purchased from online market and six lupin samples were collected from different regions of Greece, between May to July 2018–2021 (Table 4). All plant material was collected from at least three flowering individuals per population and were homogenized using a laboratory mill, transferred to properly labeled tubes, and stored at −20 °C until analysis. Seed was the studied plant part, to screen alkaloid composition of each species. All aerial parts were studied in the case of *L. pilosus* (Rethymn, Crete), collected in May 2021, and only leaves and seeds for *L. pilosus* (Mt.Athos, Chalkidiki, Macedonia), collected in July 2018–2020. All collected samples were dried at 60 °C overnight and analyzed for their QAs concentration using qNMR.

### 4.3. Quinolizidine Alkaloids Extraction and Chemical Analysis of Plant Material

QAs extraction was conducted according to the method of Wink et al. [5] with some modifications. Fine ground lupin seeds were weighted accurately (0.250 g ± 0.1 mg) and then homogenized twice with 8 mL of 0.5 N HCl in a sonicator (Semat, St Albans, UK) for 30 min. The extraction mixture was centrifuged at 4000 rpm for 5 min (Eppendorf 5810R, Hamburg, Germany), the supernatants were combined, and the pH of the solution was adjusted to 10.0 with 1 N NaOH. Afterwards, alkaloids were extracted three times, each one with 20 mL of dichloromethane. The dichloromethane extracts were combined and evaporated to dryness with a rotary evaporator (Buchi, Flawil, Switzerland) at 40 °C. The aqueous layer was then acidified with 0.5 N HCl, re-basified with 1 N NaOH to pH = 13 and re-extracted with 20 mL dichloromethane. The purpose of the second extraction was to isolate more polar alkaloids that were not extracted on the first attempt. All organic layers were combined and transferred to 50 mL round-bottom flasks, where they were mixed with 1 mL of a syringaldehyde (99% purity, Acros Organics, Steinem, Germany) solution (0.5 mg/mL) in acetonitrile (Scharlau) (as internal standard, I.S.). The mixture was evaporated to dryness, and directly subjected to quantitative analysis by NMR without any purification step.

### 4.4. ^1^H-NMR Quantitation

The residue of each sample obtained from the above-described procedure was dissolved in 750 μL deuterated chloroform (CDCl_3_) (99.80% D, Euriso-Top, Saint-Aubin, France) and the solution was transferred to a 5 mm NMR tube. ^1^H-NMR spectra were recorded at 400 MHz (Bruker Avance DRX400). Each sample was analyzed in triplicate using a standard 90-degree excitation pulse, with a pulse width of 10 s and a pre scan delay of 6.5 s. All measurements were performed at 298 K. Typically, 16 scans were collected into 32 K data points over a spectral width of 0–13 ppm (5263.18 Hz) with a relaxation delay of 10 s, an acquisition time of 3.11 s and FID resolution of 0.32 Hz. The appropriate relaxation delay was determined by gradual increases (1, 2, 5, 8, 10, 15, 20 s until the ratio between the integration of the peak of internal standard and the peak of the target compounds remained unchanged). The matching, tuning, shimming, receiver gain adjustment as well as phasing and baseline correction were always first performed automatically and then manually to achieve the best result. Prior to Fourier transformation (FT), an exponential weighting factor corresponding to a line broadening of 0.3 Hz was applied. For the peaks of interest, accurate integration was performed manually. The concentration of QAs was measured by comparing the area of the selected signal of Table 1 with that of the internal standard (IS) at 9.81 ppm, which was set as 1. The calculation of the concentration of total QAs in mg/g of dry material was performed using the following formula:

n_IS_ × I_QA_ = n_QA_ × a × I_IS_

m_QA_ = n_QA_ × MW_QA_

where,

- n_is_ = 0.0005 g/MWsyr = 0.00000274 mol
- n_QA_ = moles of each QA
- I_QA_ = integration ratio between the proton signal of syringaldehyde (IS) and the corresponding proton of the studied analyte
- I_is_ = integration of internal standard
- MW_QA_ = molecular weight of the studied analyte
- m_QA_ = mass in g of each alkaloid
- a: Coefficient depending on the integration of each peak, integrating one scale of the double peak, and integrating the entire single peak or double with a small range of Hz
- a = 1, for a single peak which integrates for a proton or for a double peak which integrates for two protons when integrating only one scale of the double peak, a = 2, for a double peak which integrates for one proton when integrating only one scale of the double peak, as in the case of multiflorine and 11,12-seco-12,13-didehydromultiflorine

The quantitation was based on the integration ratio between the proton signal of syringaldehyde at 9.81 ppm and the protons of each studied substance as shown in Table 1 and Figure 2. Non overlapping, undoubtedly defined peaks of protons were selected for quantitation. It should be noted that multiflorine was identified by integrating the one scale of the doublet peak of the proton at 6.84 ppm, while 11,12-seco-12,13-didehydromultiflorine by integrating the one scale of the double doublet peak of the proton at 6.87 ppm.

### 4.5. GC-MS Analysis

The analysis was performed following a previously established method by Przybylak et al. [11] with slight modifications. The sample extracts were analyzed using an Agilent Technologies 7820A gas chromatograph coupled with 5977B MSD mass detector. An Agilent HP-5 MS capillary column was used (30 m × 0.25 mm i.d., 0.25 μm film thickness. Helium was used as the carrier gas with a flow rate of 1.4 mL/min. The injector temperature was set at 290 °C; the detector at 300 °C. The injection was performed in a split mode, with a ratio of 1/20. The oven temperature program was isothermal at 180 °C for 2 min, then increased to 300 °C at a rate of 6 °C/min, and kept at 300 °C for 10 min. The injection volume was 1 μL. The alkaloids compounds were identified by comparison of their mass spectra with those from isolated compounds analyzed under the same conditions, and with data of the literature as well as with Wiley 275 MS library.

### 4.6. Isolation of Studied Bioactive Quinolizidine Alkaloids

Four *Lupinus* spp. extracts, i.e., *L. albus*, *L. pilosus*, *L. mutabilis* and, *L. angustifolius* were prepared according to the extraction method described above and were used for the isolation of studied metabolites using first column chromatography with silica gel (35–70 μm/Silica Flash) and then preparative thin layer chromatography (TLC) with silica gel (60 F254-Merck). The mobile phase for preparative TLC was prepared using a mixture of CHCl_3_-Acetone-diethylamine (70:20:10). TLC plates were observed under UV 254 nm and 320 nm prior to being sprayed with Dragendorff reagent solution. Mobile phase for column chromatography was CHCl_3_-MeOH-NH_4_OH solvent system of increasing polarity, and each sample was placed on column by the dry deposition technique. In this work, this technique was used to separate alkaloids of *L. albus* and *L. pilosus*, following their selective extraction from the plant. Regarding *L. angustifolius and L. mutabilis*, preparative TLC was the chosen chromatographic technique used for alkaloids isolation.

All isolated compounds were of quinolizidine core, and more specifically lupanine, sparteine, multiflorine, lupinine, angustifoline and, 11,12-seco-12,13-didehydromultiflorine. The identity of all compounds was defined by 2D-NMR study, GC-MS analysis, and comparison with literature data.

### 4.7. Resin Debittering Process

The seeds of *L. albus* (500 g) were boiled in water (1:3, seeds:water) for 75 min to destroy thermolabile anti-nutritional factors, such as trypsin inhibitors and to soften the seeds. Following boiling, the seeds were washed and placed in a container full of 1 L distilled water. Using a submersible pump (flow rate 2.5–5.0 mL/min), the water was passed through a column filled with 400 g of each of the studied resins mentioned in Table 5 and then recycled back to the container with the seeds. The pump remained in operation throughout the experiments. In all experiments the ratio of water volume to seed mass was kept constant. To prevent the alteration of the seeds by unwanted microorganisms, citric acid monohydrate was used, up to pH = 3.5.

The aqueous treatment was applied to lupin seeds with high initial alkaloids content (21.12 mg/g DM). The debittering process took 20–46 h depending on each resin.

The alkaloid levels were monitored by frequent sampling of both seeds and water. Finally, alkaloids were recovered from the anionic resins was performed with: NaOH 10% (*w*/*w*) in water or NaCl 10% (*w*/*w*) in water or water. The recovery from the adsorption resin was performed using EtOH followed by evaporation. Regarding the aqueous solutions from the anionic resins, they were extracted with CH_2_Cl_2_ which after evaporation afforded the QAs in dry form its extraction. Seed extraction after and during resin treatment was performed according to the extraction protocol described. All samples were analyzed by both GC-MS and ^1^H-NMR and quantified by ^1^H-qNMR.

#### Precision

The intraday precision (i.e., the experimental error) was determined by analyzing three replicates of three random samples in the same day and was found <8%. It was expressed as the relative standard deviation (RSD), ranged for lupanine from 1.12 to 7.27%, for multiflorine from 1.33 to 6.99%, for 11,12-seco-12,13-didehydromultiflorine from 3.20 to 6.25% and for albine from 3.75 to 6.66%. Lupinine was found only in *L. pilosus* with RSD 4.34%, while 13-OH-lupanine ranged from 2.19 to 7.54%, and angustifoline 1.80 to 7.69%. The complete intraday precision results are shown in supplementary material, Table S2.

## 5. Conclusions

As lupins are gaining outmost importance as sources of proteins, a maximum limit of total QAs content has been fixed in some countries, in order to assure the safety of lupin foods. Since the availability of commercial QA reference standards is limited, development of an efficient quantitation method is of great importance. In this context, NMR Spectroscopy was demonstrated as an analytical tool for high-throughput screening, identification, and quantitation of QAs of *Lupinus* spp. Alkaloids variations among and within *Lupinus* species were detected that could be used as analytical markers to characterize and discriminate the various species. *L. pilosus* was interestingly characterized by high amounts of almost pure multiflorine. Lupanine dominated in all *Lupinus* spp., except for *L. pilosus*, while in others, abundance of some alkaloids was characteristic for the species (e.g., angustifoline in *L. angustifolius*). Application of the developed methodology also confirmed the finding that different species of genus *Lupinus* differ from each other in terms of alkaloids content, and this may also appear inside the same species or subspecies, due to different geographic origin or/and development stage.

A lot of chemical and analytical work has been performed on QAs in order to determine chemical structures of single compounds and biochemical work to determine pharmacological or biological activities of both single compounds and complex extracts. We believe that the chemical diversity and biological activity of these compounds deserves further study to discover promising lead molecules for pharmacological applications.

Moreover, in this study, a multidisciplinary approach was explored for both the removal of alkaloids from *Lupinus* seeds, but also for the isolation of QAs from the lupin beans debittering wastewater. The effect of the aqueous debittering process on the profile and content of alkaloids was investigated in *L. albus*, which is one of the four most cultivated species worldwide. Finally, we showed that the debittering process through the continuous use of ionic or adsorption resin can be significantly accelerated and simplified in combination with the rapid analysis of QAs with qNMR.

## Figures and Tables

**Table 1:** Molecular weights, structures, and selected signals (ppm) which can be used for quantitation of QAs in natural extracts of *Lupinus* sp.

Quinolizidine Alkaloid	Structure	Proton Signal	δ in ppm
Lupanine	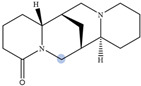	H10 eq	4.49
13-OH-lupanine	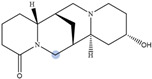	H10 eq	4.58
Sparteine	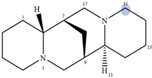	H15 eq	2.80
Multiflorine	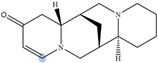	H2	6.84
11,12-seco-12,13-didehydromultiflorine	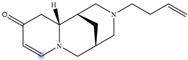	H2	6.87
Angustifoline	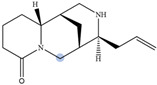	H10 eq	4.66
Albine	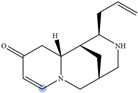	H2	6.92
Lupinine	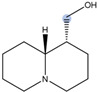	H11	4.12

The blue circle in each molecule indicates the position of the protons that were used for quantitation.

**Table 2:** Concentrations of QAs in extracts of the seeds of each of the studied species, expressed in mg/g DW.

Species (Origin)	Lupanine		Multiflorine		Albine		Angustifoline	
	Mean	S.D.	Mean	S.D.	Mean	S.D.	Mean	S.D.
*L. pilosus*, Mt. Athos	nd	nd	6.83	0.09	nd	0.00	nd	nd
*L. pilosus*, Crete	5.81	0.12	2.33	0.06	0.15	0.01	0.13	0.01
*L. albus*, Sparta	22.55	0.36	2.49	0.05	1.31	0.09	0.74	0.03
*L. albus*, Alexandroupoli	12.17	0.22	1.16	0.06	0.37	0.02	0.30	0.02
*L. albus*, Crete	15.51	0.18	2.43	0.17	0.80	0.03	0.97	0.06
*L. albus var. sweet*, Larisa	nd	nd	0.16	0.01	nd	0.00	nd	nd
*L. angustifolius*	3.16	0.23	nd	nd	nd	nd	1.00	0.02
*L. mutabilis*	7.33	0.47	nd	nd	nd	nd	nd	nd
*L. elegans*	4.44	0.05	nd	nd	nd	nd	nd	nd
*L. luteus*	nd	nd	nd	nd	nd	nd	nd	nd
*L. perennis*	5.60	0.29	nd	nd	nd	nd	2.22	0.04
*L. nanus*	3.90	0.22	nd	nd	nd	nd	nd	nd
*L. polyphyllus*	6.82	0.37	nd	nd	nd	nd	0.78	0.03
*L. hartwegii*	4.57	0.17	nd	nd	nd	nd	nd	nd
*L. cruckshankii*	2.93	0.13	nd	nd	nd	nd	nd	nd
**Species (Origin)**	**13-OH-Lupanine**		**11,12-seco-12,13** **Didehydromultiflorine**		**Lupinine**		**Sparteine**	
	**Mean**	**S.D.**	**Mean**	**S.D.**	**Mean**	**S.D.**	**Mean**	**S.D.**
*L. pilosus*, Mt. Athos	nd	nd	nd	nd	0.23	0.01	nd	nd
*L. pilosus*, Crete	0.16	0.01	0.16	0.01	nd	nd	nd	nd
*L. albus*, Sparta	2.75	0.07	0.55	0.02	nd	nd	nd	nd
*L. albus*, Alexandroupoli	0.86	0.05	0.28	0.01	nd	nd	nd	nd
*L. albus*, Crete	2.73	0.06	0.62	0.02	nd	nd	nd	nd
*L. albus var. sweet*, Larisa	nd	nd	nd	nd	nd	nd	nd	nd
*L. angustifolius*	nd	nd	nd	nd	nd	nd	nd	nd
*L. mutabilis*	0.23	0.01	nd	nd	nd	nd	6.92	0.22
*L. elegans*	nd	nd	nd	nd	nd	nd	ov	nd
*L. luteus*	nd	nd	nd	nd	nd	nd	nd	nd
*L. perennis*	0.53	0.04	nd	nd	nd	nd	nd	nd
*L. nanus*	nd	nd	nd	nd	nd	nd	ov	nd
*L. polyphyllus*	0.37	0.02	nd	nd	nd	nd	nd	nd
*L. hartwegii*	nd	nd	nd	nd	nd	nd	ov	nd
*L. cruckshankii*	nd	nd	nd	nd	nd	nd	2.43	0.18

nd: not detected, ov: overlapped, S.D. = standard deviation.

**Table 3:** Concentrations of QAs in extracts of *L. pilosus* aerial parts, expressed in mg/g DW.

Plant Part	Lupanine		Multiflorine		Albine	
	Mean	S.D.	Mean	S.D.	Mean	S.D.
flower	nd	0.00	7.21	0.09	nd	0.00
leaf	0.12	0.01	3.59	0.11	nd	0.00
pod	3.56	0.12	6.12	0.11	0.13	0.01
seeds	5.81	0.12	2.33	0.06	0.15	0.01
stem	1.02	0.07	nd	0.00	nd	0.00
**Plant Part**	**Angustifoline**		**13-OH-Lupanine**		**11,12-seco-12,13** **Didehydromultiflorine**	
	**Mean**	**S.D.**	**Mean**	**S.D.**	**Mean**	**S.D.**
flower	nd	0.00	nd	0.00	nd	0.00
leaf	nd	0.00	nd	0.00	nd	0.00
pod	0.20	0.01	0.12	0.01	0.18	0.01
seeds	0.13	0.01	0.16	0.01	0.16	0.01
stem	nd	0.00	nd	0.00	nd	0.00

nd: not detected, S.D. = standard deviation.

**Table 4:** Sampling region and plant part of the studied *Lupinus* spp.

Species	Origin	Series	Plant Part
*L. albus*	Crete	wild	seeds
*L. albus*	Alexandroupoli	wild	seeds
*L. albus*	Sparta	wild	seeds
*L. albus* var. *sweet*	Larisa	cultivated	seeds
*L. pilosus*	Rethymn, Crete	wild	flower, leaf, pod, steam, seeds
*L. pilosus*	Mt. Athos, Northeastern Greece	wild	seeds, leaf
*L. angustifolius*	England	cultivated	seeds
*L. cruckshankii*	England	‘Sunrise,’ Paysons lupin, Paradox lupine	seeds
*L. elegans*	England	‘Pink Fairy,’ Mexico lupin	seeds
*L. hartwegii*	England	‘Avalune Red White,’ Dwarf lupin	seeds
*L. mutabilis* var. *cruckshankii*	England	‘Javelin White’	seeds
*L. nanus*	England	‘Snow Pixie,’ Dwarf lupin, Sky lupin	seeds
*L. perennis*	Mexico	wild	seeds
*L. polyphyllus*	England	‘Band of Nobles’ series, ‘Noble Maiden’	seeds
*L. luteus*	England	cultivated	seeds

**Table 5:** Resins used for debittering process of *L. albus* seeds.

Resin Type	Resin Characteristics
Amberlite^®^ XAD7HP, 20–60 mesh (St. Louis, MO, USA)	nonionic, aliphatic acrylic polymer
Amberlite^®^ IRC120 H, hydrogen form (St. Louis, MO, USA)	strongly acidic cation exchange resin
Purolite^®^ C100E, Ionic Form (Na^+^ form) (St. Louis, MO, USA)	Polystyrenic Gel, Strong Acid Cation Resin

## Data Availability

Data are contained within the article and Supplementary Materials.

## Supplementary Materials

The following supporting information can be downloaded at: https://www.mdpi.com/article/10.3390/molecules29030582/s1, Figure S1: Quantitation results of QAs concentration in extracts of each of the studied species, expressed in mg/g DW. Figure S2: ^1^H-NMR spectra (400 MHz, CDCl_3_, δ-values in ppm) from all *Lupinus* spp. quantified. Figure S3: ^1^H-NMR spectra (400 MHz, CDCl_3_, δ-values in ppm) from all aerial parts of *L. pilosus* from Crete in an early stage of development. Table S1: Characteristic peaks from 1H-NMR spectrum of studied alkaloids and their δ-values in ppm, used for quantitation. Table S2: Intraday precision data for the studied substances, expressed as relative standard deviations (%).
