# Supplementary material for: Quantitation of Lupinus spp. Quinolizidine Alkaloids by qNMR and Accelerated Debittering with a Resin-Based Protocol
Source: Molecules. 2024 Jan 24;29(3):582. doi: 10.3390/molecules29030582 (PMC10856427; doi:10.3390/molecules29030582)
Supplement: Supplementary file 1 [file molecules-29-00582-s001.zip › molecules-2814342-supplementary.pdf]

molecules

Article

Quantitation of *Lupinus* spp. Quinolizidine Alkaloids by qNMR and Accelerated Debittering with a Resin-Based Protocol

Nikoleta Anna Madelou, Eleni Melliou, Prokopios Magiatis*

Department of Pharmacy, Laboratory of Pharmacognosy and Natural Products Chemistry, National and Kapodistrian University of Athens, 15771 Athens, Greece; nikoletamadelou@gmail.com (N.M); emel-liou@pharm.uoa.gr (E.M.); magiatis@pharm.uoa.gr (P.M.)

Table S1. Characteristic peaks from 1H-NMR spectrum of studied alkaloids and their δ-values in ppm, used for quantitation.

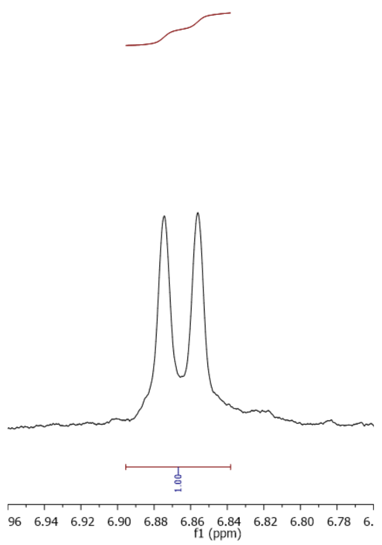	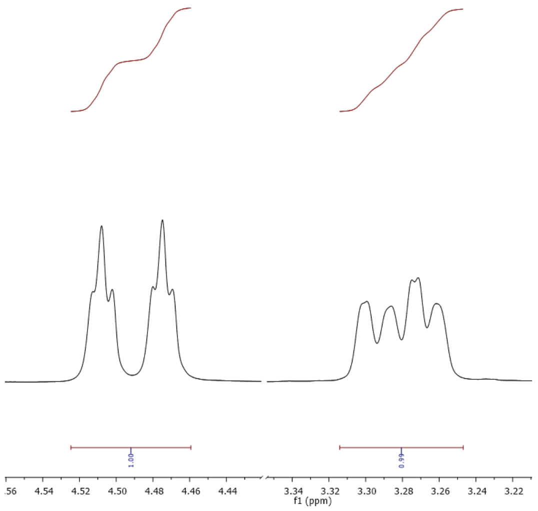
11,12-seco-12,13 didehydromultiflorine	Lupanine

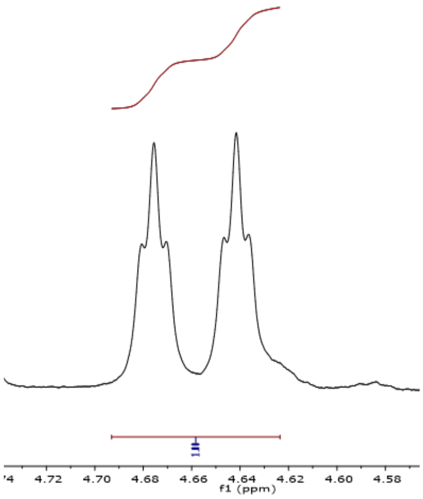

Angustifoline

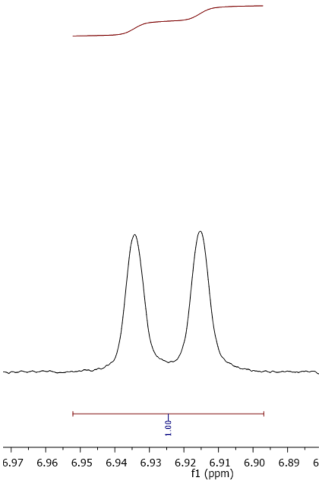

Albine

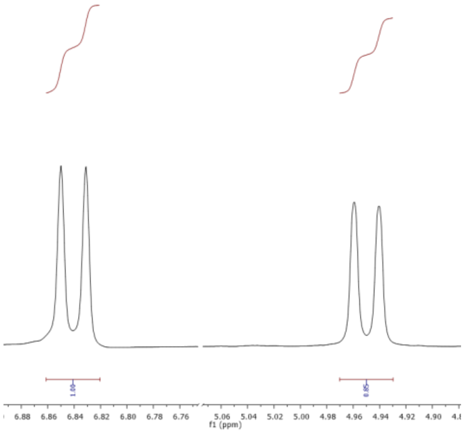

Multiflorine

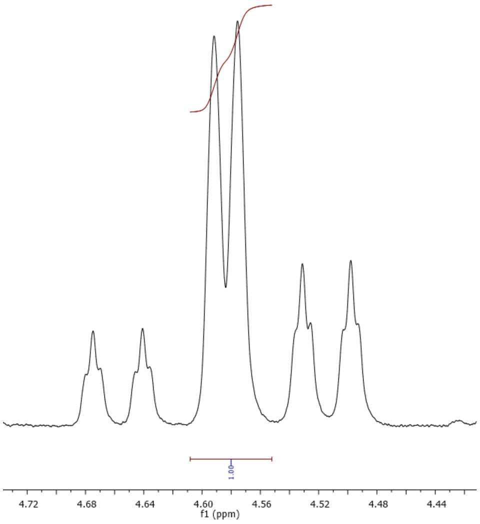

13-OH-lupanine

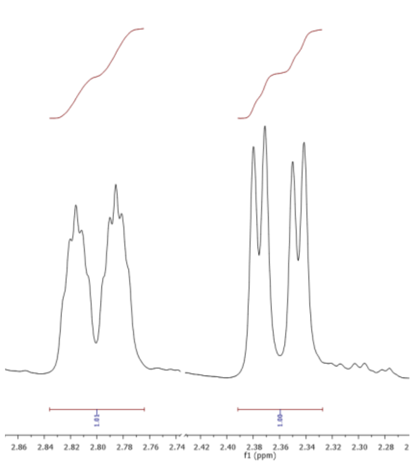	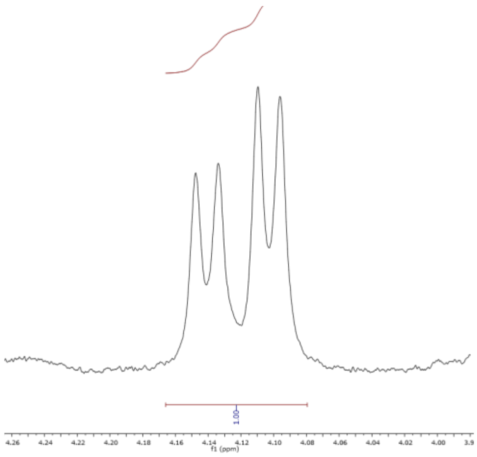
Sparteine	Lupinine

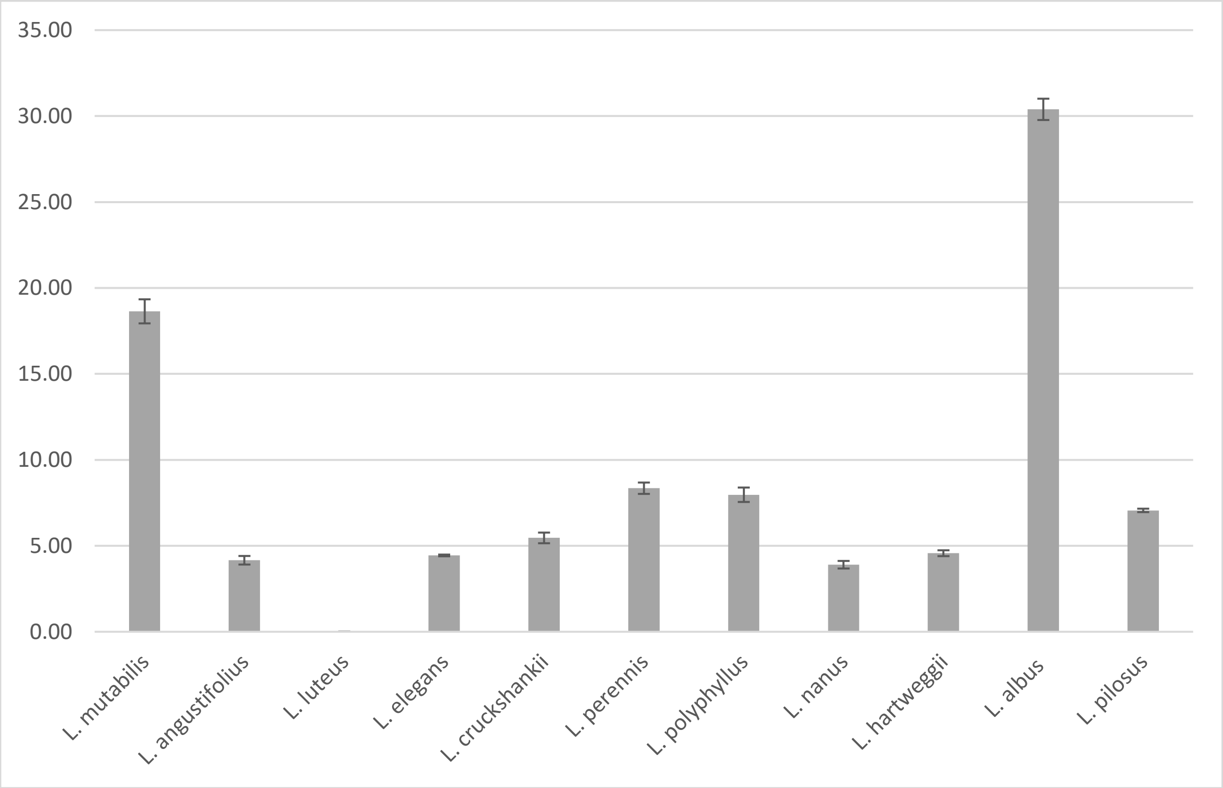

Figure S1. Quantitation results of QAs concentration in extracts of each of the studied species, ex-pressed in mg/g DW.

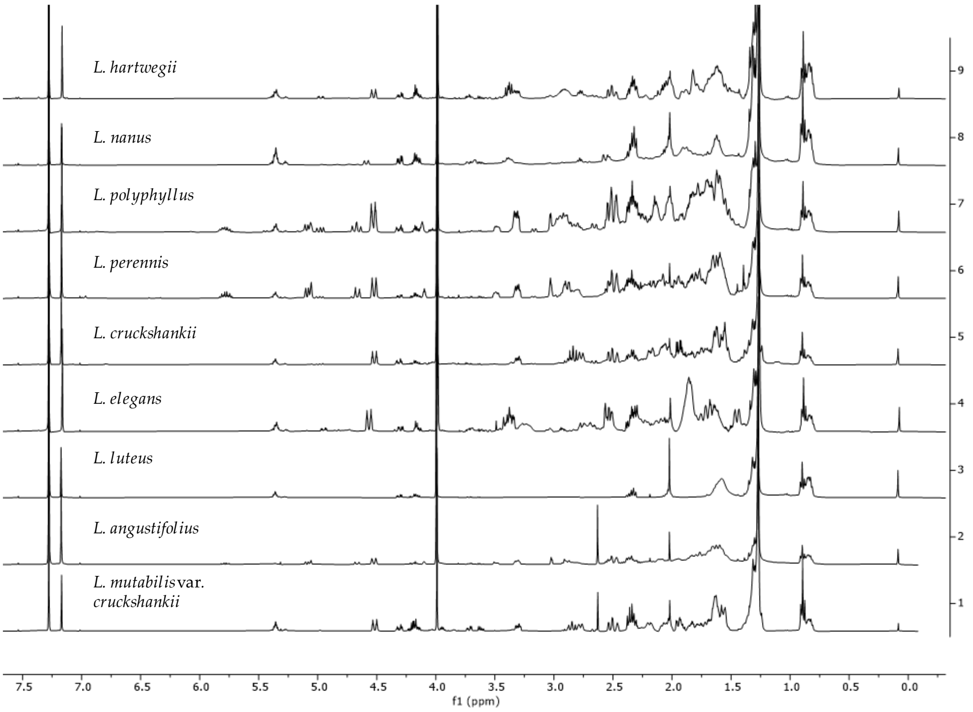

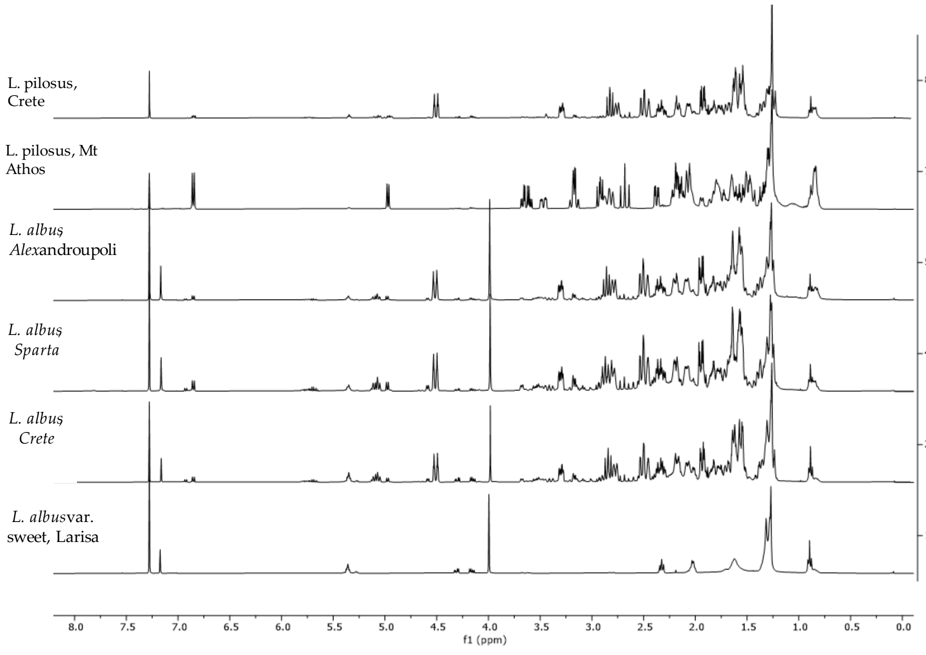

Figure S2. ^{1}H-NMR spectra (400 MHz, $CDCl_3$, δ-values in ppm) from all *Lupinus* spp. quantified.

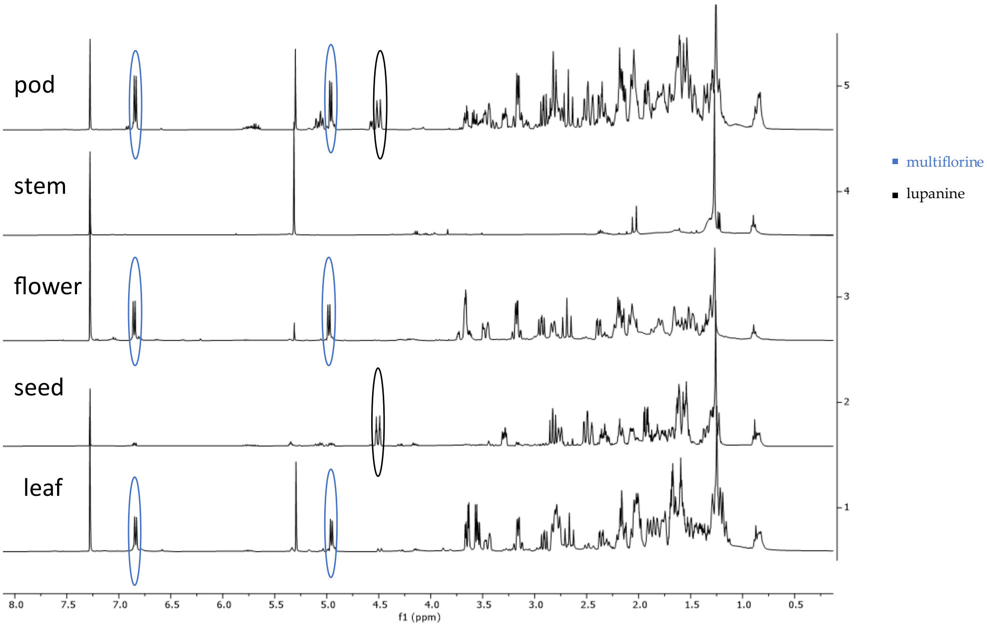

Figure S3. ^{1}H-NMR spectra (400 MHz, $CDCl_3$, δ-values in ppm) from all aerial parts of *L. pilosus* from Crete in an early stage of development.

Table S2. Intraday precision data for the studied substances, expressed as relative standard deviations (%).

Species	N	Lupanine	13-OH-lupanine	Multiflorine	Lupinine
L. albus, Crete	3	1.16	2.19	6.99	-
L. albus, Alexandroupoli	3	1.80	5.81	5.17	-
L. albus, Sparta	3	1.59	2.54	2.00	-
L. albus var. sweet	3	-	-	6.25	-
L. pilosus Crete	3	2.06	6.25	2.57	-
L. pilosus, Mt. Athos	3	-	-	1.33	4.34
L. angustifolius	3	7.27	-	-	-
L. cruckshankii	3	4.43	-	-	-
L. elegans	3	1.12	-	-	-
L. hartwegii	3	3.71	-	-	-
L. mutabilis var. cruckshankii	3	6.41	4.34	-	-
L. nanus	3	5.64	-	-	-
L. perennis	3	5.17	7.54	-	-
L. polyphyllus	3	5.42	5.40	-	-
L. luteus	3	-	-	-	-

Species	N	11,12-seco-12,13-didehydromulti-florine	Angustifoline	Albine	Sparteine
L. albus, Crete	*3*	3.20	6.18	3.75	-
L. albus, Alexandroupoli	*3*	3.57	6.66	5.40	-
L. albus, Sparta	*3*	3.63	4.05	6.87	-
L. albus var. *sweet*	*3*	-	-	-	-

L. pilosus Crete	*3*	6.25	7.69	6.66	-
L. pilosus, Mt. Athos	*3*	-	-	-	-
L. angustifolius	*3*	-	2.00	-	-
L. cruckshankii	*3*	-	-	-	7.40
L. elegans	*3*	-	-	-	-
L. hartwegii	*3*	-	-	-	-
L. mutabilis var. *cruckshankii*	*3*	-	-	-	3.17
L. nanus	*3*	-	-	-	-
L. perennis	*3*	-	1.80	-	-
L. polyphyllus	*3*	-	3.84	-	-
L. luteus	*3*	-	-	-	-
